# Brown Adipose Tissue Rescues Bone Loss Induced by Cold Exposure

**DOI:** 10.3389/fendo.2021.778019

**Published:** 2022-01-20

**Authors:** Jingke Du, Zihao He, Mingming Xu, Xinhua Qu, Junqi Cui, Shuangyan Zhang, Shuhong Zhang, Hanjun Li, Zhifeng Yu

**Affiliations:** ^1^ Shanghai Key Laboratory of Orthopedic Implants, Department of Orthopedic Surgery, Shanghai Ninth People’s Hospital, Shanghai Jiao Tong University School of Medicine, Shanghai, China; ^2^ Knee Surgery Department of the Institute of Sports Medicine, Beijing Key Laboratory of Sports Injuries, Peking University Third Hospital, Beijing, China; ^3^ Arthritis Clinic and Research Center, Peking University People’s Hospital, Peking University, Beijing, China; ^4^ Department of Bone and Joint Surgery, Renji Hospital, School of Medicine, Shanghai Jiao Tong University, Shanghai, China; ^5^ Department of Pathology, Shanghai Ninth People’s Hospital, Shanghai Jiao Tong University School of Medicine, Shanghai, China

**Keywords:** cold exposure, bone remodeling, osteoblast, osteoclast, interleukin-6

## Abstract

Cold temperature activates the sympathetic nervous system (SNS) to induce bone loss by altering bone remodeling. Brown adipose tissue (BAT) is influenced by the SNS in cold environments. Many studies have confirmed a positive relationship between BAT volume and bone mass, but the influence and mechanism of BAT on bone *in vivo* and *in vitro* is still unknown. Two-month-old C57/BL6j male mice were exposed to cold temperature (4°C) to induce BAT generation. BAT volume, bone remodeling and microstructure were assessed after 1 day, 14 days and 28 days of cold exposure. CTX-1, P1NP and IL-6 levels were detected in the serum by ELISA. To determine the effect of BAT on osteoclasts and osteoblasts *in vitro*, brown adipocyte conditional medium (BAT CM) was collected and added to the differentiation medium of bone marrow-derived macrophages (BMMs) and bone marrow mesenchymal stem cells (BMSCs). Micro-CT results showed that the bone volume fraction (BV/TV, %) significantly decreased after 14 days of exposure to cold temperature but recovered after 28 days. Double labeling and TRAP staining *in vivo* showed that bone remodeling was altered during cold exposure. BAT volume enlarged after 14 days of cold stimulation, and IL-6 increased. BAT CM promoted BMSC mineralization by increasing osteocalcin (Ocn), RUNX family transcription factor 2 (Runx2) and alkaline phosphatase (Alp) expression, while bone absorption was inhibited by BAT CM. In conclusion, restoration of bone volume after cold exposure may be attributed to enlarged BAT. BAT has a beneficial effect on bone mass by facilitating osteogenesis and suppressing osteoclastogenesis.

## Introduction

The body’s metabolism can be affected by many factors, such as food intake (1, 2), exercise (3, 4), stress state (5, 6), and environmental temperature (7). Low-temperature exposure can affect the activity of the nervous system (8–10), endocrine system (11, 12), musculoskeletal system (13–15) and so on (7, 16). All of these factors influence the expression levels of cytokines *in vivo*. The relationship between temperature and bone mass has triggered researchers’ interest in recent years, but the effects of cold exposure on bone have not been well illustrated. Some results declared that cold leads to increased bone mass (13), while other studies indicated that cold has negative effects on bone volume (14, 17). Studies have shown that cold exposure affects the generation and release of neurotransmitters, which play a role in skeletal metabolism and endocrinology (10, 18). Wee, N.K.Y., et al. confirmed that neuropeptide Y (NPY) plays a significant role in the increase in energy expenditure, UCP1 expression, and bone loss in response to cold exposure (10). Low temperature-activated sympathetic nerves lead to the activation of β adrenergic receptors and subsequently initiate osteoclast-related bone resorption (19, 20). In addition, activated sympathetic nerves can influence bone mass in indirect ways by affecting the expression levels of bone morphogenetic protein 8b (BMP8b) and PTH (20, 21).

In addition to its effects on bone mass, cold stimulation also promotes the generation of brown adipose tissue (BAT) and increased UCP1 expression levels (22, 23), which is essential for brown adipocytes. Similar to white adipose tissue, BAT can be considered an endocrine organ that secretes many factors under both physiological and pathological conditions (24, 25). These adipose cytokines have widespread functions. In addition to its influence on lipid metabolism, BAT releases fibroblast growth factor 21 (FGF21), interleukin-6 (IL-6) (26), and neuregulin 4 (Nrg4) to influence metabolism (27, 28). Reports have shown that brown adipokines, such as BMP8b and Nrg4, influence the remodeling of the neurovascular network and alleviate liver steatosis (29, 30). Enlarged BAT *via* hypertrophy and hyperplasia (31) increases energy consumption and leads to increased bone mass (32).

The positive relationship between BAT and bone mass has been previously confirmed (33). Bredella, M. A. et al. found that BAT has a positive effect on bone mass and that BAT is a positive predictor of femoral bone structure (34). Bone formation ability was attenuated in BAT-deficient mice (*Misty* mice) (35). Correlation analyses in humans showed that BAT is an independent predictor of bone mass (13, 16). In this study, we aimed to investigate the effect and mechanism of BAT on bone metabolism, which could help to elucidate the functions and applications of BAT.

## Materials and Methods

### Animals

Male C57BL/6J mice at two months of age were purchased from Shanghai SLAC Laboratory Animal Company (Shanghai, China), and this study was approved by the animal ethics committee of Shanghai Ninth People’s Hospital. The mice were fed commercial food and water under specific aseptic (SPF) conditions. For cold stimulation, thirty mice were randomly divided into two groups, cold stimulation (cold) and room temperature (normal), with fifteen mice per group. Briefly, mice in the cold group grew in incubators (Fuyilian, FYL-YS-280 L) at 4°C, while mice in the normal group were in the same type of incubators at room temperature (23°C) (36). Mice were euthanized after being exposed to cold/normal temperature for 1 d, 14 d and 28 d, and tibias were then collected.

### Calcein and Alizarin Red Double Labeling

To calculate dynamic bone histomorphometry, animals were injected with 30 mg/kg calcein (Sigma) and alizarin red (Sigma) 10 and 3 days before euthanasia. Nondecalcified tibiae were embedded in methyl methacrylate and sectioned.

After imaging with a confocal microscope, histomorphometric examination was confined to the consistent cortical region and was performed using BIOQUANT OSTEO 2019 (v19.6.60). The mineral apposition rate (MAR) and bone formation rate per bone surface (BFR/BS) were analyzed at 40× magnification from 6 representative fields per bone sample (37).

### Microcomputed Tomography Scanning

At the end of each experiment, the tibias of the mice were fixed in 4% paraformaldehyde. Samples were scanned using micro-CT (μCT 80; Scanco, Zurich, Switzerland) as previously described (38). The micro-CT parameters were as follows: voltage, 70 kV; electric current, 114 μA; and resolution, 10 μm per pixel. Three-dimensional structural parameters, including bone volume fraction (BV/TV), trabecular number (Tb.N), trabecular thickness (Tb.Th) and trabecular separation (Tb.Sp), were analyzed (38, 39).

### *In Vivo* Brown Adipose Volume Analysis

Perkin Elmer micro-CT was applied to analyze brown adipose tissue as described in previous studies (40, 41). Briefly, mice were anesthetized with 30 mg/kg pentobarbital sodium and then scanned in the machine to measure BAT volume in the scapula. The data were analyzed using Analyze 12.0 according to the instructions to determine the BAT volume.

### Fat Mass and Lean Mass Measurement

Mice were scanned by dual-emission X-ray absorptiometry (DXA, Hologic Discovery A) through animal models to obtain the lean mass, %lean, fat mass, %fat and whole-body weight.

### Enzyme-Linked Immunosorbent Assay (ELISA)

Interleukin-6 (IL-6) (70-EK206/3-96, Multisciences), P1NP (Lengton, Shanghai), and CTX-1 (Lengton, Shanghai) ELISA kits were used to detect the expression levels of IL-6, P1NP, and CTX-1 in the serum, according to the manufacturer’s instructions. A microplate reader (Bioteck, Arcugnano [Vicenza], Italy) was used to determine the optical density (OD) of each well at 450 nm.

### TRAP Staining

After decalcification in 10% ethylenediaminetetraacetic acid (EDTA) for 3 weeks, samples were embedded in paraffin. To observe the microstructure of the samples, 4-µm-thick sagittal sections of the medial compartment of the knee joint were cut. After TRAP staining was performed, OC surface/bone surface (Oc.N/BS) was calculated using a Bioquant system.

### *In Vitro* Differentiation of BAT

BAT was isolated and cultured following previously described methods (42). Briefly, 4-week-old C57BL/6 mice were euthanized. Interscapular BAT was collected, minced, and digested in collagenase digestion buffer (DMEM, 1 mg/ml collagenase I, 1% FBS). Preadipocyte cells were collected by filtering through 70 µm membranes and centrifuging. Preadipocytes were cultured to 80%-90% confluence in DMEM supplemented with 10 ng/ml bFGF (Pepro Tech), 10% fetal bovine serum (Gibco) and pen/strep (Life Technologies). Cells were subcultured every 3 days and used from passages 3 to 5. Then, preadipocytes were differentiated with DMEM containing 10% fetal bovine serum (Gibco), 10 µg/ml insulin (Sigma), 1 µM dexamethasone (Sigma), 0.5 mM 3-isobutyl-1-methylxanthine, phosphodiesterase inhibitor (IBMX, Sigma), 5 µM rosiglitazone (Sigma), and 1 nM T3 (Sigma) for 6 days until brown adipocyte formation.

### Preparation of BAT CM

To obtain conditioned medium (BAT CM), DMEM with 10% exosome-free FBS was used to culture brown adipocytes, which were collected after 48 hours. The conditioned medium was centrifuged at 300 × g for 10 min to discard cells and further centrifuged at 2,000 × g for 10 min and at 10,000 × g for 30 min to remove cellular debris and large vesicles, respectively (43, 44). After filtration through a 0.22 µm filter, conditioned medium was used to culture BMMs and BMSCs.

### *In Vitro* Osteoclastogenesis

To obtain bone marrow macrophages (BMMs), 4-week-old C57BL/6J mice were sacrificed, and their femurs and tibias were separated under sterile conditions. Bone marrow was flushed from the mouse femurs and tibias using complete a-MEM, which contained macrophage-colony stimulating factor (M-CSF, 30 ng/mL). After resuspension, bone marrow cells were cultured in a 10-cm dish at 37°C in 5% CO_2_. The medium was changed after 3 and 7 days to remove nonadherent cells. When the cellular density reached 80% confluence, BMMs were washed three times with phosphate buffered saline (PBS) and collected using 0.25% trypsin for subsequent experiments. Bone mesenchymal stem cells (BMSCs) were obtained through the same procedure but were cultured without M-CSF (45). For osteoclastogenesis, BMMs were seeded into 24-well plates (10^5^ cells/well) or 96-well plates (10^4^ cells/well) and then treated with osteoclastogenesis medium, which consisted of complete a-MEM, receptor activator of nuclear factor kappa-B ligand (RANKL, 50 ng/mL) and M-CSF (30 ng/mL). BAT CM was added or not to the osteoclastogenesis medium for 7 days until osteoclasts were formed (46). After fixing the cells in 4% paraformaldehyde for 30 min, TRAP-positive cells were stained using a TRAP staining kit (Sigma-Aldrich, 387A-1KT). ImageJ software (Media Cybernetics Bethesda, MD, United States) was used to calculate the number of multinuclear (n≥3) TRAP-positive cells in each well. To determine the bone resorption ability, BMMs were differentiated in an osteoassay stripwell plates for 8 days. Then, the cells were washed with 4% sodium hypochlorite for 10 minutes followed by three washes with double distilled water. The resorption area was calculated using ImageJ software (Media Cybernetics Bethesda, MD, United States).

### *In Vitro* Osteogenesis

BMSCs were seeded into 24-well plates and cultured until the they reached 80% confluence. Then, the medium was replaced with an osteogenesis assay kit (MUBMX-90021, Cyagen, CA, United States) with or without BAT conditioned medium (1:1) at 37°C in humidified air with 5% CO_2_ for 21 days to induce osteogenesis. Bone formation was detected using alkaline phosphatase (ALP) or alizarin red staining on days 14 and 21. ALP staining was performed as follows: after washing three times with PBS and fixation in 4% paraformaldehyde for 10 min at room temperature, cultured cells were stained using the BCIP/NBT Alkaline Phosphatase Color Development Kit (Beyotime Institute of Biotechnology, Shanghai, China). All steps were strictly in accordance with the manufacturer’s instructions. After 21 days of culture, alizarin red staining was performed. Briefly, the cultured cells were washed with PBS and fixed in 4% paraformaldehyde for 30 min, and then 500 µL alizarin red dye (contained in the MUBMX-90021 kit) was added to each well and incubated at room temperature for 10 min. After washing five times with PBS, 10% cetylpyridinium chloride (500 µL) (H811089, Macklin, CA, United States) was added to each well for semiquantitative analysis. Then, the absorbance of the supernatant at 562 nm was detected after incubation for 30 min at room temperature.

### Quantitative Reverse‐Transcription Polymerase Chain Reaction (qRT-PCR)

TRIzol reagent (Thermo Scientific, US) was used to extract total RNA. After the concentration was measured, the total RNA was converted to complementary DNA using a Quant script RT Kit (Promega, Madison, WI, USA). To detect messenger RNA (mRNA) levels, cDNA and SYBR Premix Ex Taq Mix (Selleck) PCR in 10 µL PCRs were performed in the Real‐Time PCR System (Light Cycler 2.0; Roche Diagnostics GmbH, Mannheim, Germany). The primer sequences are shown in **Table 1**.

**Table 1:** Primer sequences for the quantitative reverse‐transcription polymerase chain reaction.

Target genes	Forward (5’-3’)	Reverse (5’-3’)
Gapdh	AGGTCGGTGTGAACGGATTTG	TGTAGACCATGTAGTTGAGGTCA
Ocn	CTGACCTCACAGATCCCAAGC	TGGTCTGATAGCTCGTCACAAG
Runx2	CCGGGAATGATGAGAACTA	ACCGTCCACTGTCACTTT
Alp	CCAACTCTTTTGTGCCAGAGA	GGCTACATTGGTGTTGAGCTTTT
Traf6	AAAGCGAGAGATTCTTTCCCTG	ACTGGGGACAATTCACTAGAGC
Ctsk	GAAGAAGACTCACCAGAAGCAG	TCCAGGTTATGGGCAGAGATT
Atp6a3	CACAGGGTCTGCTTACAACTG	CGTCTACCACGAAGCGTCTC
Dcst	GGGGACTTATGTGTTTCCACG	ACAAAGCAACAGACTCCCAAAT
UCP1	AGGCTTCCAGTACCATTAGGT	CTGAGTGAGGCAAAGCTGATTT
Pgc1α	TATGGAGTGACATAGAGTGTGCT	CCACTTCAATCCACCCAGAAAG
Cidea	TGACATTCATGGGATTGCAGAC	GGCCAGTTGTGATGACTAAGAC
Prdm16	CCAAGGCAAGGGCGAAGAA	AGTCTGGTGGGATTGGAATGT

### Statistical Analysis

GraphPad Prism 5.0 (GraphPad Software Inc., CA, United States) was used for statistical analysis of data. Each experiment was repeated at least three times. For animal studies, each group had at least three mice. All quantitative values are presented as the mean ± standard deviation (SD). Two−way analysis of variance (ANOVA) or Student’s t-test was used for analysis of differences. Bonferroni correction was performed for multiple comparisons. P < 0.05 was considered statistically significant.

## Results

### Cold Exposure Influences Bone Mass in a Time-Dependent Manner

**Figure 1:**
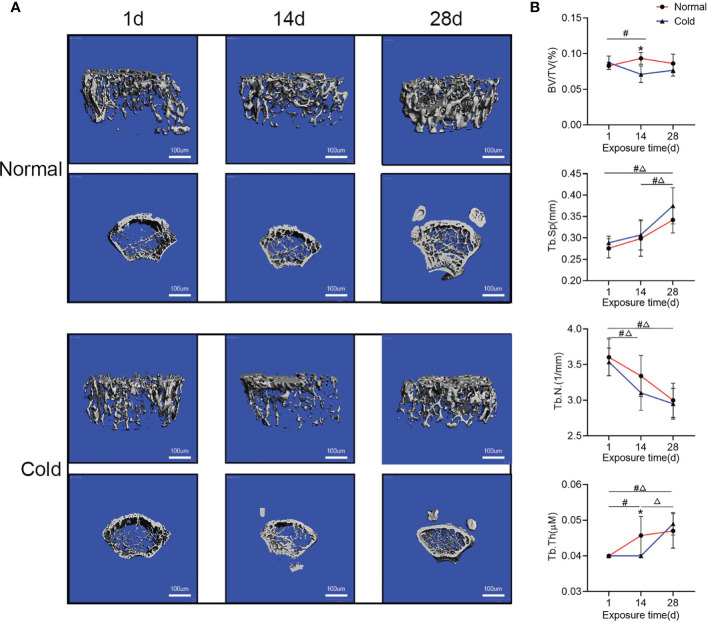
Bone mass fluctuates with increased time. **(A)** Representative images of micro-CT are shown. **(B)** Trabecular bone microarchitecture of tibias showing bone volume/total volume (BV/TV), trabecular number (Tb.N), trabecular separation (Tb.Sp), and trabecular thickness (Tb.Th). Data are shown as the mean ± SD (n=5 per group). Significance (p-value) was calculated using two-way ANOVA, *p < 0.05, cold group *vs*. normal group; ^#^p < 0.05, difference at different time points in the normal group; ^Δ^p < 0.05, difference at different time points in the cold group; 1 day (1 d), 14 days (14 d), 28 days (28 d), Normal: mice at room temperature (23°C), Cold: mice at 4°C.

After being exposed to 4°C for different times, mice exhibited fluctuations in bone microstructure (**Figure 1A**). As shown in **Figure 1B**, BV/TV and Tb.Th decreased at 14 days but recovered after 28 days of exposure. Tb.Sp in both groups increased after 28 days of exposure, but it was higher in the cold-treated group. Tb.N in both groups decreased with prolonged time and was lower in the cold-treated group.

### Cold Exposure Affects Bone Remodeling

**Figure 2:**
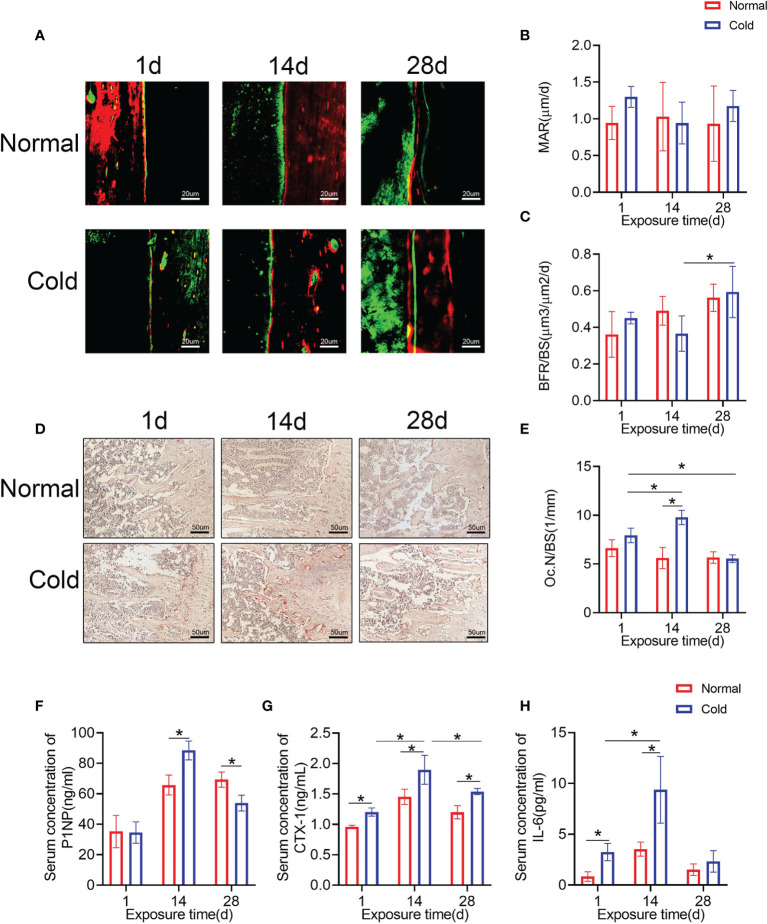
Bone remodeling is influenced by temperature. **(A)** Calcium and alizarin red double labeling were performed to determine the bone formation rate. **(B, C)** BFR/BS and MAR shown in A were calculated. **(D)** Representative TRAP staining of femur bone sections. **(E)** Histomorphometric quantification of Oc.N/BS in femur bone. **(F–H)** CTX-1, P1NP and IL-6 in the serum were detected by ELISA. Black arrows: TRAP positive cells. Data are shown as the mean ± SD (n=5 per group). Significance (p-value) was calculated using two-way ANOVA, *p < 0.05; 1 day (1 d), 14 days (14 d), 28 days (28 d), Normal: mice at room temperature (23°C), Cold: mice at 4°C.

Calcein and alizarin red double labeling were performed to determine the bone formation rate (**Figure 2A**). As shown in **Figure 2C**, the bone formation rate/bone surface (BFR/BS) decreased dramatically after 14 days of cold stimulation and increased at 28 days, but there were no significant changes in the mineral appositional rates (MARs) (**Figure 2B**). Osteoclast numbers were calculated using TRAP staining (**Figure 2D**), and increased osteoclast number/bone surface (Oc.N/BS) of the cold group was observed at 14 days (**Figure 2E**), which was recovered even lower than the baseline after 28 days of exposure. Levels of P1NP in the cold group, a marker of bone formation, were higher at 14 days and lower at 28 days than that in the control group (**Figure 2F**). CTX-1 was detected in the same way to confirm bone resorption ability. Expression levels of CTX-1 in the cold group were higher than that in the control group and reached their highest level at 14 days (**Figure 2G**). In addition, cold promoted the expression of IL-6, one of the most important factors induced by BAT (26), at 1 and 14 days (**Figure 2H**).

### Brown Adipose Tissue Accumulates in a Cold Environment *In Vivo*

**Figure 3:**
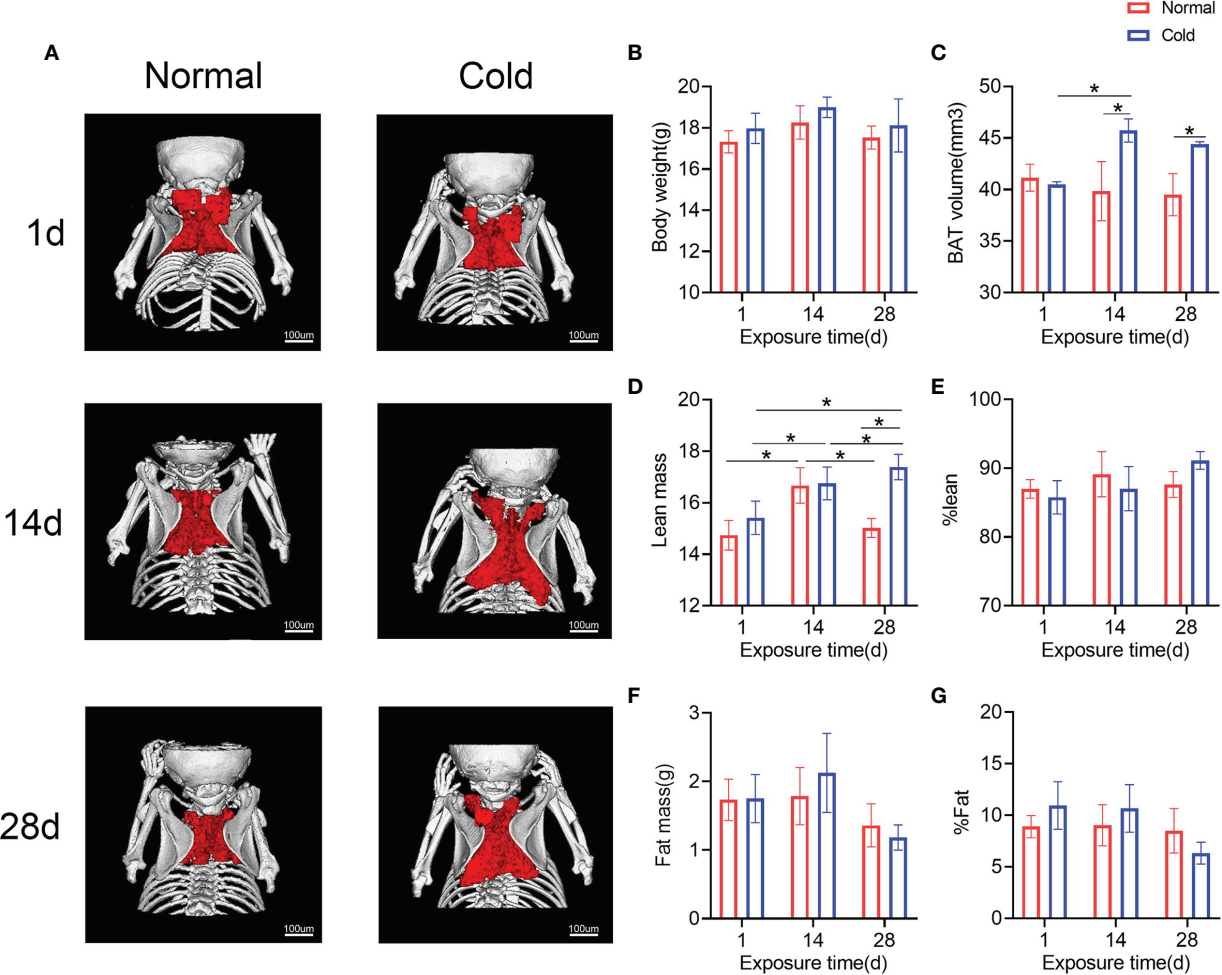
Brown adipose tissue in the interscapula enlarges in response to cold exposure. **(A)** Reconstruction images of the micro-CT are shown, brown adipose reconstructs with red and bone with white. **(B–G)** Body weight, brown adipose volume (BAT volume), lean mass, %lean, fat mass and %fat were calculated. Data are shown as the mean ± SD (n=5 per group). Significance (p-value) was calculated using two-way ANOVA, *p < 0.05; 1 day (1 d), 14 days (14 d), 28 days (28 d), Normal: mice at room temperature (23°C), Cold: mice at 4°C.

To observe the effect of cold on BAT, BAT in the interscapular region of mice was scanned and calculated. As shown in **Figures 3A, C**, brown adipose volume was stimulated by cold temperature, which increased in 14 days and was maintained until 28 days. Concordantly, the lean mass in both groups was higher on day 14 than on day 1, while the cold group exhibited a higher lean mass on day 28 (**Figure 3D**). Other results calculated by DXA, such as body weight, fat mass, %lean and %fat, did not change significantly (**Figures 3B**, **E**–**G**).

### Brown Adipocytes Affect Osteogenesis and Osteoclastogenesis *In Vitro*

**Figure 4:**
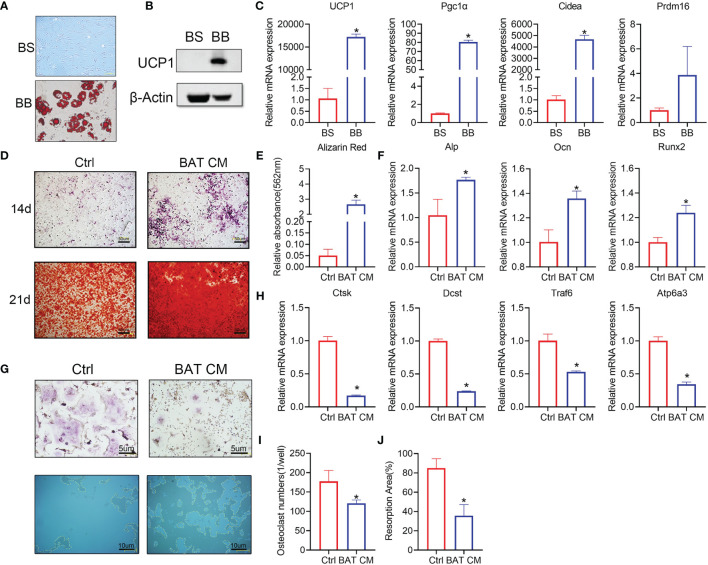
Brown adipocytes affect bone remodeling *in vitro*. **(A)** Oil red O staining was performed to show the distribution of lipid droplets in brown adipocytes. **(B)** Western blot analysis showed that UCP1 expression was higher in the differentiation group. **(C)** RT-PCR shows the relative mRNA expression levels of the two groups. **(D)** ALP and alizarin red staining were performed at 14 and 21 days. **(E)** Semiquantitative analysis of alizarin red staining. **(F)** Alp, Ocn and Runx2 expression levels are shown. **(G)** TRAP staining (top) and bone resorption ability (below) in the hydroxyapatite covered plate are shown. Absorbed areas are circled with yellow dotted lines. **(H)** Relative expression levels of Ctsk, Dcst, Traf6, and Atp6a3 were detected by RT-PCR. **(I)** Osteoclast numbers in each well are calculated. **(J)** The osteoclast resorption area in the osteoassay stripwell plate was computed. Data are shown as the mean ± SD (n≥3 per group). Significance (p-value) was calculated using Student’s t-test, *p < 0.05; 1 day (1 d), 14 days (14 d), 28 days (28 d), Normal: mice at room temperature (23°C), Cold: mice at 4°C.

Brown adipose tissue cells in the stromal vascular fraction (BAT svf, BS) were cultivated *in vitro*. Oil red O staining was performed to test whether BAT SVF cells were differentiated into brown adipocytes (BB) (**Figure 4A**), which produce scattered lipid droplets. Western blot analysis showed that UCP1 expression was increased in the BB group (**Figure 4B**). Furthermore, brown adipocyte gene markers were dramatically increased in the differentiated group (**Figure 4C**).

Brown adipocyte conditional medium (BAT CM) was added to the osteogenesis differentiation medium of BMSCs. Staining assays showed that BAT CM strengthened ALP and alizarin red staining in BMSCs (**Figure 4D**). Semiquantitative analysis of alizarin red is shown in **Figure 4E**, which confirmed improved bone formation in the BAT CM group. Moreover, Alp, Ocn, and Runx2 levels were upregulated by BAT CM compared to the corresponding control treatments (**Figure 4F**).

To determine the effects of BAT CM on osteoclastogenesis, we added BAT CM to the differentiation medium of BMMs, and TRAP staining (**Figure 4G**) revealed reduced multinuclear osteoclastogenesis after 6 days of cultivation (**Figure 4I**). Meanwhile, resorption ability was assessed using the osteoassay stripwell, and the resorption area was smaller in the BAT CM group (**Figure 4J**). Additionally, Ctsk, Dcst, and Traf6 were downregulated by BAT CM (**Figure 4H**).

## Discussion

In this research, fluctuating bone mass was observed in response to the extension of cold exposure time, decreased at 14 days, and increased at 28 days. Further detection revealed that there was increased bone formation and osteoclast numbers at 14 days. Studies *in vitro* showed that BAT CM promoted osteogenesis and impaired osteoclastogenesis.

Cold-induced bone loss after 14 days of exposure is consistent with research showing that low temperature is negatively correlated with bone mass. Robbins et al. found that cool nursed mice exhibited reduced bone mass but higher UCP1 expression at 20°C versus 26°C (17). Similarly, growing C57BL/6J and C3H/HeJ mice nursing at 22°C resulted in premature cancellous bone loss (14, 47). In addition, Serrat et al. excluded the effect of tissue perfusion on extremity elongation in mice and attributed shorter hindlimbs to alterations in chondrocyte proliferation and extracellular matrix volume in the cold environment (48). Other study concluded that cold has positive effect on bone mass. They performed their studies in anorexia nervosa people and cold stimulation was used as a tool to select people with brown adipose, and then the positive relationship between brown adipose volume and bone mass was concluded (13). In our study, cold induced bone loss after 14 days of exposure, which is consistent with research showing that low temperature is negatively correlated with bone mass.

As the regulator of bone remodeling, the sympathetic nervous system can be activated by exercise, cold, and emotion (19, 20). The negative relationship between the activation of sympathetic nerves and bone mass has been confirmed (49). As a sympathetic nerve enrichment organ (50, 51), cold-induced enlargement of BAT is well defined (28). Recently, Moser C et al. reported that cold promotes the generation of BAT after 1 week of exposure, which is consistent with our results (52). It is generally acknowledged that brown adipocytes consume energy and have a wide range of functions (24, 25, 31). An important role of BAT in bone remodeling has been confirmed in FoxC2(AD)(+/Tg) mice, which exhibit enhanced bone remodeling ability and increased bone mass (37). In another study, Katherine J Motyl et al. raised *Misty* mice, brown adipose barren mice, in a cold (4°C) environment and concluded that a short period of cold stimulation decreases RUNX2 and increases RANKL expression levels (35). In addition, brown adipocytes play a role in BMP-2-induced heterotopic ossification in muscle (53).

Besides, BAT can secrete cytokines, such as leptin, fibroblast growth factor 21(FGF21), IL-6, and neuregulin 4, all of them could influence bone remodeling (27, 54). Leptin, a well-studied protein, has direct anabolic effect on osteoblast (55), and BMSCs rich in leptin receptor (LepR) are the main source of bone formation (56). Studies in healthy adults found a positive association between plasma FGF21 levels and BMD in women (57), while Ruo-Han Hao et al. observed that FGF21 is negatively related to regional BMD in humans (58). Inconsistent results were also reported in animal studies (59, 60). So, it is reasonable to assume that these adipokines play a role in the changes of bone mass under cold stress. Similarly, IL-6 may play a role in the process of bone mass fluctuation during cold exposure. Reports have confirmed that mice are under stress in cold environments (61, 62), which leads to the release of IL-6 by BAT (26). All of these findings indicate that low temperature may accelerate bone renewal by promoting osteogenesis and osteoclastogenesis. As a well-defined cytokine, IL-6 is a type of interleukin that can be produced by fibroblasts, macrophages, T lymphocytes, B lymphocytes, endotheliocytes, keratinocytes, and a variety of tumor cells (63) and has a wide range of functions *in vivo (* 64). In terms of its effects on bone remodeling, it is believed that IL-6 enhances osteoclast differentiation (65, 66), but it does not promote osteoclastogenesis in a direct way, even in osteoclasts or their progenitor cells expressing the IL-6 receptor (67). In contrast, like many other cytokines and hormones, IL-6 indirectly promotes osteoclast formation and bone resorption by promoting RANKL expression (64). For example, IL-6 promotes expression of RANKL in osteoblast cell lines *in vivo (* 68, 69), thereby activating osteoclasts to promote bone resorption. Studies *in vivo* found that ovariectomy-induced bone loss can be alleviated by inhibiting the activity of IL-6, indicating the obvious effect of IL-6 on the activity of osteoclasts (70). In our study, IL-6 expression levels were markedly increased on day 14 of cold stimulation but gradually decreased with the extension of exposure time, accompanied by changes in the number of osteoclasts, which increased on day 14 and decreased on day 28. Therefore, it can be speculated that bone loss caused by a low-temperature environment after 14 days is closely related to IL-6 and controlling the expression level of IL-6 may represent a potential target for osteoporosis treatment. Considering the significant effects of cytokines mentioned above, BAT may promote osteogenesis *in vivo and vitro* by secreting cytokines or extracellular vesicles.

In recent years, increasing attention has been given to the role of small extracellular vesicles in information transmission. It has been reported that fibroblasts of young human donors alleviate certain senescence biomarkers of cells derived from old donors (71). Similarly, small extracellular vesicles derived from osteoclasts or tumor cells can influence bone formation or resorption (72, 73). Furthermore, miRNAs have been found to be secreted extracellularly in exosomes and to have a wide range of functions (74). For example, miRNA-5106 in M2 macrophage-derived exosomes accelerates fracture healing *in vivo (* 75). In addition, miRNA-21 and miRNA-217 can transmit senescence signals to neighboring endothelial cells (76). Moreover, studies found that brown adipose depletion impairs bone remodeling *in vivo (* 35), while brown adipocyte-derived exosomes alleviate metabolic syndrome in high-fat diet mice (77). Therefore, we postulate that the bone rescue ability of BAT CM is attributed to the exosomes contained in it, and further, some microRNAs may be identified as promoting these effects.

In conclusion, the changes in bone mass that occur in low temperature conditions may be the result of a combination of temperature and BAT. The exact mechanism may be complicated, but it is clear that BAT secretes a number of factors that influence bone mass. The striking influence of BAT CM on bone formation and osteoclastogenesis indicates that further studies should be performed to detect the important factors in this process, which could represent an important treatment for osteoporosis. In summary, the functional cytokines or extracellular vesicles in BAT CM need further investigation.

## Data Availability Statement

The raw data supporting the conclusions of this article will be made available by the authors, without undue reservation.

## Ethics Statement

The animal study was reviewed and approved by Animal Ethics Committee of Shanghai Ninth People’s Hospital.

## Author Contributions

All authors listed have made a substantial, direct, and intellectual contribution to the work, and approved it for publication.

## Funding

This work was supported by grants from the National Natural Science Foundation of China (Nos. 11572197, 11872251, 81802679) and the National Key R&D Program (grant no. 2016YFC1102100).

## Conflict of Interest

The authors declare that the research was conducted in the absence of any commercial or financial relationships that could be construed as a potential conflict of interest.

## Publisher’s Note

All claims expressed in this article are solely those of the authors and do not necessarily represent those of their affiliated organizations, or those of the publisher, the editors and the reviewers. Any product that may be evaluated in this article, or claim that may be made by its manufacturer, is not guaranteed or endorsed by the publisher.
